# Field-grown miR156 transgenic switchgrass reproduction, yield, global gene expression analysis, and bioconfinement

**DOI:** 10.1186/s13068-017-0939-1

**Published:** 2017-11-30

**Authors:** Chelsea R. Johnson, Reginald J. Millwood, Yuhong Tang, Jiqing Gou, Robert W. Sykes, Geoffrey B. Turner, Mark F. Davis, Yi Sang, Zeng-Yu Wang, C. Neal Stewart

**Affiliations:** 1 Department of Plant Sciences, University of Tennessee, Knoxville, TN USA; 2 BioEnergy Science Center, Oak Ridge National Laboratory, Oak Ridge, TN USA; 3 Noble Research Institute, Ardmore, OK USA; 4 National Renewable Energy Laboratory, Golden, CO USA

**Keywords:** Bioconfinement, Floral transition, miR156, Switchgrass, Gene flow

## Abstract

**Background:**

Genetic engineering has been effective in altering cell walls for biofuel production in the bioenergy crop, switchgrass (*Panicum virgatum*). However, regulatory issues arising from gene flow may prevent commercialization of engineered switchgrass in the eastern United States where the species is native. Depending on its expression level, microRNA156 (miR156) can reduce, delay, or eliminate flowering, which may serve to decrease transgene flow. In this unique field study of transgenic switchgrass that was permitted to flower, two low (T14 and T35) and two medium (T27 and T37) miR156-overexpressing ‘Alamo’ lines with the transgene under the control of the constitutive maize (*Zea mays*) ubiquitin 1 promoter, along with nontransgenic control plants, were grown in eastern Tennessee over two seasons.

**Results:**

miR156 expression was positively associated with decreased and delayed flowering in switchgrass. Line T27 did not flower during the 2-year study. Line T37 did flower, but not all plants produced panicles. Flowering was delayed in T37, resulting in 70.6% fewer flowers than controls during the second field year with commensurate decreased seed yield: 1205 seeds per plant vs. 18,539 produced by each control. These results are notable given that line T37 produced equivalent vegetative aboveground biomass to the controls. miR156 transcript abundance of field-grown plants was congruent with greenhouse results. The five miR156 SQUAMOSA PROMOTER BINDING PROTEIN-LIKE (*SPL*) target genes had suppressed expression in one or more of the transgenic lines. Line T27, which had the highest miR156 overexpression, showed significant downregulation for all five *SPL* genes. On the contrary, line T35 had the lowest miR156 overexpression and had no significant change in any of the five *SPL* genes.

**Conclusions:**

Because of the research field’s geographical features, this study was the first instance of any genetically engineered trait in switchgrass, in which experimental plants were allowed to flower in the field in the eastern U.S.; USDA-APHIS-BRS regulators allowed open flowering. We found that medium overexpression of miR156, e.g., line T37, resulted in delayed and reduced flowering accompanied by high biomass production. We propose that induced miR156 expression could be further developed as a transgenic switchgrass bioconfinement tool to enable eventual commercialization.

**Electronic supplementary material:**

The online version of this article (doi:10.1186/s13068-017-0939-1) contains supplementary material, which is available to authorized users.

## Background

Switchgrass (*Panicum virgatum* L.) is a native North American perennial prairie grass mostly known for its use as a biofuel feedstock. The high biomass production, low input requirements, and its ability to be productive on marginal land are some features that make switchgrass an attractive cellulosic feedstock [1, 2]. However, the high degree of lignification of secondary cell walls (around 20% of switchgrass dry cell wall biomass) inhibits biomass conversion to fermentable sugars and biofuel in switchgrass, which, in turn, is an economic barrier to biofuel production [1–5]. Genetic engineering to reduce lignin levels in switchgrass cell walls appears to be essential for its optimal use as a biofuel crop [6–8]. Indeed, there are several success stories in producing transgenic switchgrass with altered lignification, which resulted in higher biofuel yield from field-grown biomass (e.g., [10, 11]), but the prospects of transgene flow from genetically engineered switchgrass is a regulatory concern. Transgene flow from switchgrass will likely need to be severely curtailed to facilitate the commercialization of transgenic varieties [6, 9]. This situation is especially pertinent in the eastern United States where switchgrass is endemic and common [12]. Research has investigated several bioconfinement strategies, which include pollen ablation [13–15] and removal via site-specific recombinases [16, 17]. In addition, the delay or elimination of flowering itself could promote simultaneous improvements for a transgenic biomass crop such as switchgrass: it could decrease or eliminate pollen while simultaneously increase vegetative biomass [8, 18].

MicroRNAs (miRNAs) are an extensive class of small (20–24 nucleotides) regulatory RNAs that could be useful in genetic engineering to improve biofuel feedstocks by targeting stress responses, biomass production, and lignin content [19–31]. Specifically, miR156 targets the SQUAMOSA PROMOTER BINDING PROTEIN-LIKE (*SPL*) transcription factor family, which is involved in the transition from vegetative to reproductive phases [32–35]. Overexpression of miR156 in switchgrass at low and moderate levels led to increased biomass and a non-flowering phenotype in the greenhouse [36]. When two low and two moderate overexpressing lines were grown in the field, three of the lines flowered and one of these lines produced more biomass than the control [37]. These results indicate that growth environment and gene expression play significant roles in the phenology of switchgrass. It should also be noted that the overexpression of miR156 at moderate to high levels led to an increase in saccharification efficiency and reduction in lignin content [36, 37].

Our research objectives in this study were to deploy a range of miR156-overexpressing switchgrass in a relevant field situation to closely examine flowering, reproduction, and biomass production. A field that would be considered a ‘marginal’ site (soils, fertility, and slope) on the Cumberland Plateau in Tennessee that is surrounded by forest enabled a 2-year study in which U.S. regulators allowed switchgrass plants to reproduce. In assessing a delayed/decreased flowering strategy for transgene bioconfinement of switchgrass, it was imperative to obtain two full flowering cycles in the field to gauge the degree of practical utility of this strategy. A transcriptomic study of the field-grown plants was performed to assess the influence of downstream genes impacted by miR156 expression, as well as any potential off-target effects, which are important for designing next-generation transgenic plants to further fine-tune the spatio-temporal expression of miR156 in switchgrass.

## Methods

### Field design and plant materials

**Fig. 1:**
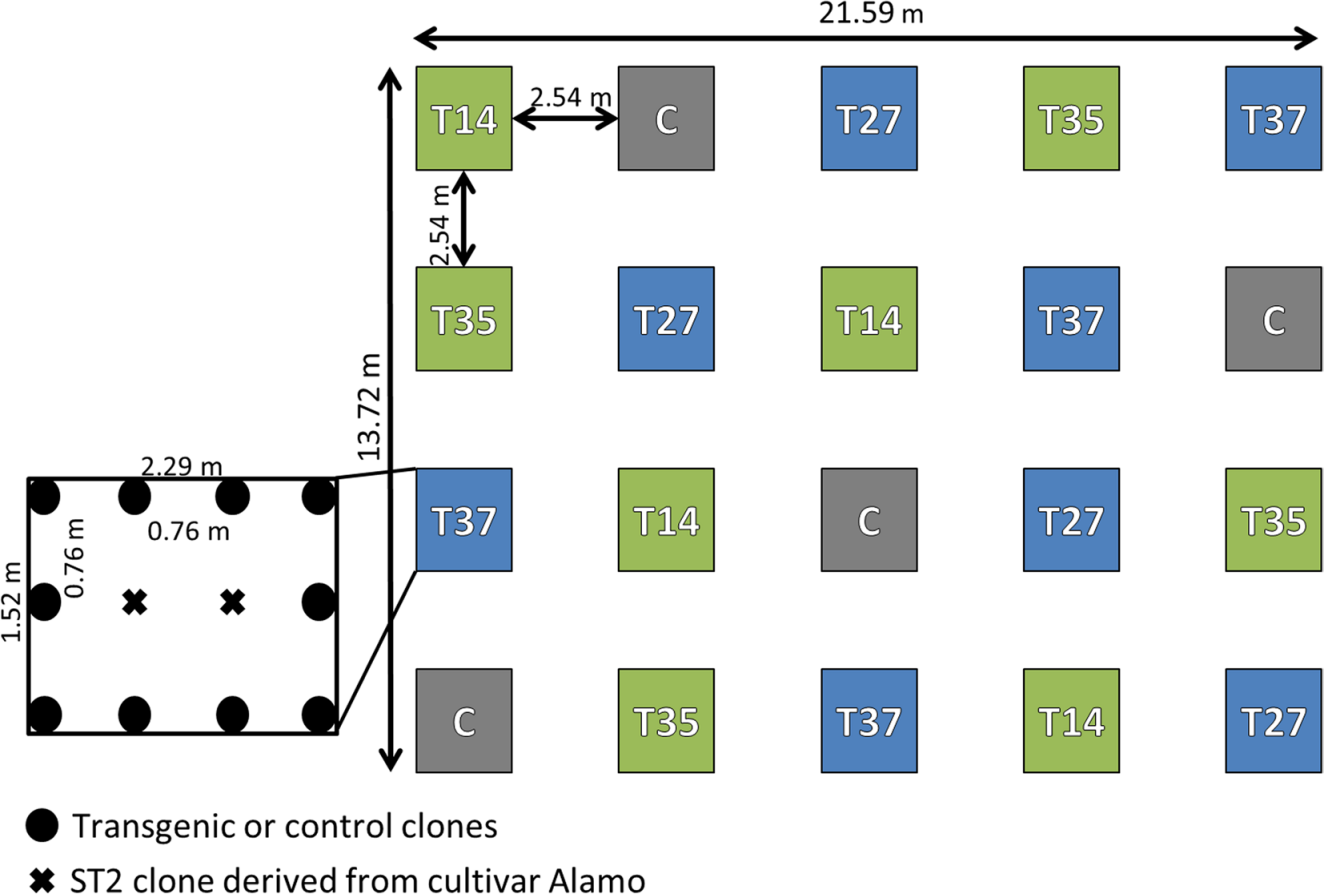
Complete randomized field design for open-flowering miR156-overexpressing transgenic switchgrass in Oliver Springs, TN, USA. In each of the 20 plots, two ‘Alamo’ ST2 clones (X’s) act as pollen donors for the surrounding 10 clones (filled black circles) from a single transgenic line (T14, T35, T27, or T37) or the ‘Alamo’ control (C). Low overexpression lines are labeled in green, and medium overexpression plots are in blue

Plants were grown in a field site in Oliver Springs, Tennessee, USA for 2 years under USDA-APHIS-BRS release permits (13-046-104r-a1 and 16-056-103r). This highly secluded field on the hilly Cumberland Plateau is surrounded by a natural forest border (Additional file 1: Figure S1), which allowed for open flowering and seed production of the transgenic switchgrass lines under permit conditions. The switchgrass plants were transplanted on June 5, 2015 into a twenty-plot complete randomized design (Fig. 1; Additional file 1: Figure S1). Four transgenic and two nontransgenic parent ‘Alamo’ switchgrass lines were used to comparatively examine the phenotypic effects of miR156 overexpression (Additional file 1: Figure S1). The four transgenic lines were engineered to overexpress the rice (*Oryza sativa*) *pre*-*miR156b* gene under the control of the maize (*Zea mays*) Ubi1 promoter as described in Fu et al. [36] at relatively low (lines T14 and T35) or medium (lines T27 and T37) overexpression levels. All transgenic plant replicates were clones obtained through vegetative propagation of tillers from the respective transgenic event. Each of the deployed lines was clonally replicated in the greenhouse prior to field transplantation. Two replicates of a second nontransgenic clone (ST2) were included as pollen donors for the surrounding ten clones representing single lines per plot (Fig. 1). Within plots, plants were spaced 0.76 m from each other, and each plot measured 2.29 m × 1.52 m. The entire field site was 21.59 m × 13.72 m. Plants were hand watered for four weeks after establishment. No fertilizer or pesticide treatments were applied during the experiment. Weeds were manually removed.

### Biomass and morphological characterization

Plants were checked weekly for the presence of panicles during both growing seasons, and first-date-to-flower was recorded. Aboveground biomass was harvested 10 cm above soil level after first frost (November) with plots pooled into a single harvest bag; the two ST2 plants from each plot were bagged separately from the surrounding plants per plot. All harvested biomass was oven-dried at 40 °C for 168 h, then dry biomass was tallied on a per-plot basis, and data were presented on a per-plant basis. Panicles were removed prior to harvest due to permit restrictions and bagged separately. Bags were stored in a greenhouse and allowed to air dry. Total panicle weights were recorded, averaged, and added to the average vegetative biomass weight to give total aboveground biomass production.

Panicles were counted during the removal process, and the lengths were measured for two randomly chosen panicles from each of five randomly selected plants per plot. A subsample of three panicles at the R4 stage of reproduction [38] was collected in September 2016 (year two) from each plot to tally flowers and spikelets per panicle.

The number of tillers per plant was tallied at each end-of-season harvest. Plant height (apex) was measured both before and after panicle removal. Leaf length, leaf width, stem diameter, and node number were taken at the end of the season on the two tallest tillers of each plant sampled. Leaf blade length and width were taken on the flag leaf or topmost mature leaf of each of the selected tillers. Tiller node number was counted from the soil line up, and representative internode diameter was taken using a Maxwell 150-mm digital caliper between the third and fourth nodes.

### Seed collection and germination

After mature seeds were harvested from panicles, three subsamples per plant were tallied for 100-seed weight, then averaged. Seed number per plant was then derived by bulk seed weight and 100-seed weight. Seeds collected from transgenic lines or nontransgenic ‘Alamo’ controls were placed on solid MS basal medium [39], and germination percentage was calculated at 2 weeks after plating.

### Cell wall characterization

End-of-season vegetative dry biomass was chipped to approximately 10-cm segments using a CS-4325 chipper shredder (Troy-Bilt, Valley City, Ohio) and then milled with a Wiley mill (Thomas Scientific, Model 4, Swedesboro, N.J.) through a 1-mm screen. Milled material was used to analyze the lignin content, syringyl-to-guaiacyl (S/G) monolignol ratio, and sugar release of the cell walls of each line by the National Renewable Energy Laboratory standard protocols. Lignin content and the S/G ratio were determined by pyrolysis molecular beam mass spectrometry as described in Sykes et al. [40] on an Extrel single-quadrupole molecular beam mass spectrometer. The peak intensities of lignin precursors were summed and used to estimate total lignin content. The S/G ratio was calculated by dividing the intensity of the syringyl peaks by the intensity of the guaiacyl peaks.

Sugar release was determined using the methods described in Selig et al. [41]. Hydrolysis took place using the Ctec2 enzyme cocktail (Novozymes North America, Franklinton, NC). Released glucose levels were measured using the d-Glucose Assay Kit (glucose oxidase/peroxidase; GOPOD), and released xylose levels were determined by the d-Xylose Assay Kit (xylose dehydrogenase; XDH; Megasyme Intl., Bray, Ireland). Sugar release data were reported as grams of released sugar per gram of cell wall residue.

### Transcriptomic analysis

Microarray analysis was performed to determine downstream gene expression effects of miR156 overexpression. Three tillers were collected from each plot, resulting in four biological replicates for each of the four transgenic and ‘Alamo’ nontransgenic control lines. Total RNA was extracted from the combined tissues of randomly selected V3 stage tillers, as defined in Hardin et al. [42], from each line harvested on September 10, 2015 between 11:00 a.m. and 1:00 p.m. RNA was extracted using Tri-Reagent (Invitrogen, Carlsbad, Calif.) and subsequently cleaned and concentrated with the RNeasy^®^ MinElute Cleanup Kit (Qiagen, Valencia, Calif.). Purified RNA (100 ng) was used for the expression analysis of each sample using a custom-designed switchgrass cDNA chip Pvi_cDNAa520831 (Affymetrix, Santa Clara, CA). Probe labeling, chip hybridization, and scanning were performed according to the manufacturer’s instructions for 3′ IVT PLUS Kit (Affymetrix). Data normalization among chips was conducted using the robust multichip average (RMA) [43]. Gene selections based on Associative *T* test [44] were made using Matlab (MathWorks, Natick, MA). In this method, the background noise presented among replicates and technical noise during microarray experiments were measured by the residual presented among a group of genes whose residuals are homoscedastic. Genes whose residuals between the compared sample pairs that are significantly higher than the measured background noise level were considered to be differentially expressed. A selection threshold of 2 for transcript ratios and a Bonferroni-corrected *P* value threshold of 5.84201E−07 were used. The Bonferroni-corrected *P* value threshold was derived from 0.05/*N* in these analyses, where *N* is the number of probe sets on the chip. Microarray data are available in the ArrayExpress database accession number E-MTAB-5948 (http://www.ebi.ac.uk/arrayexpress).

Quantitative RT-PCR (qRT-PCR) analysis was used to assess transcript abundance of miR156 and its known target *SPL* genes. Total RNA was extracted using Tri-Reagent (Invitrogen) from V3 stage tillers collected mid-day on July 26, 2016. RNA samples were cleaned with the RNeasy^®^ Mini Kit (Qiagen). The mature miR156 levels were determined using a highly sensitive stem-loop pulsed reverse transcription procedure [45] using a miR156-specific stem-loop primer. RT-PCR for *SPL* expression was performed using the High Capacity cDNA Reverse Transcription Kit (Applied Biosystems, Foster City, Calif.). SYBR Green (Applied Biosystems) was used as the reporter dye during qRT-PCR, and a QuantStudio™ 6 Flex Real-Time PCR System (Applied Biosystems) was used. The miR156 target gene transcript abundance qRT-PCR analysis included *PvSPL1, PvSPL2, PvSPL3,* and *PvSPL6*. miR156 expression was normalized using miR390 expression, and switchgrass *PvUbq1* transcript abundance was used for normalization of data from each target gene with appropriate primers [36]. Delta cycle threshold (ΔC*t*) was calculated by subtracting the target gene C*t* from the C*t* of the housekeeping gene (Housekeeping C*t*—Target C*t* = ΔC*t*).

### Statistical analysis

SAS version 9.4 (SAS Institute Inc., Cary, NC) was used for all statistical analyses. A one-way ANOVA with Fisher’s least significant difference was used to compare means among lines within each year. Differences were considered significant when *P* values were less than or equal to 0.05.

## Results

### miR156 overexpression levels affect flowering timing and reproductive effort

**Fig. 2:**
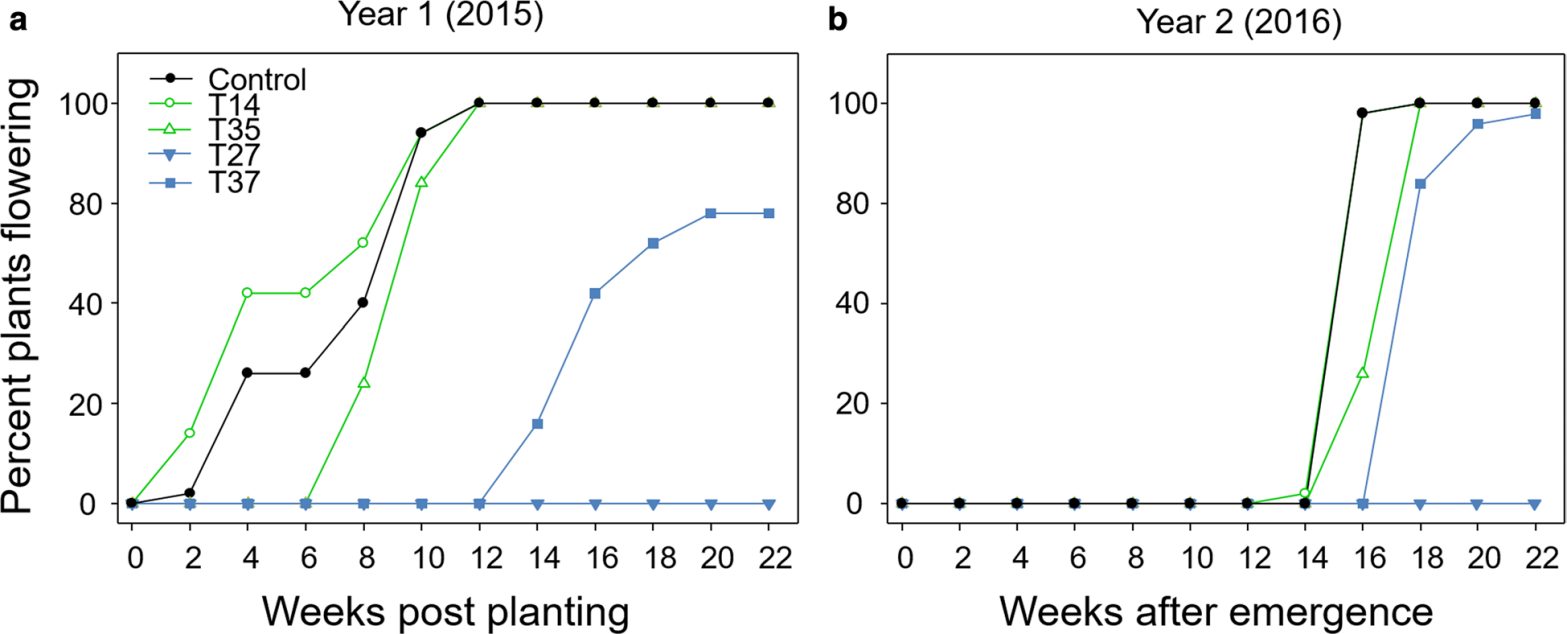
Time to first flower in the field for miR156 transgenic switchgrass lines and wild-type control. **a** Year one (2015) weeks to first panicle emergence for each line after planting on June 05, 2015 (week 0). **b** Year two (2016) weeks to first flower for each line after plant vegetative growth began on March 30, 2016 (week 0). Note that the T14 data in **b** follow the control data after week 14

**Fig. 3:**
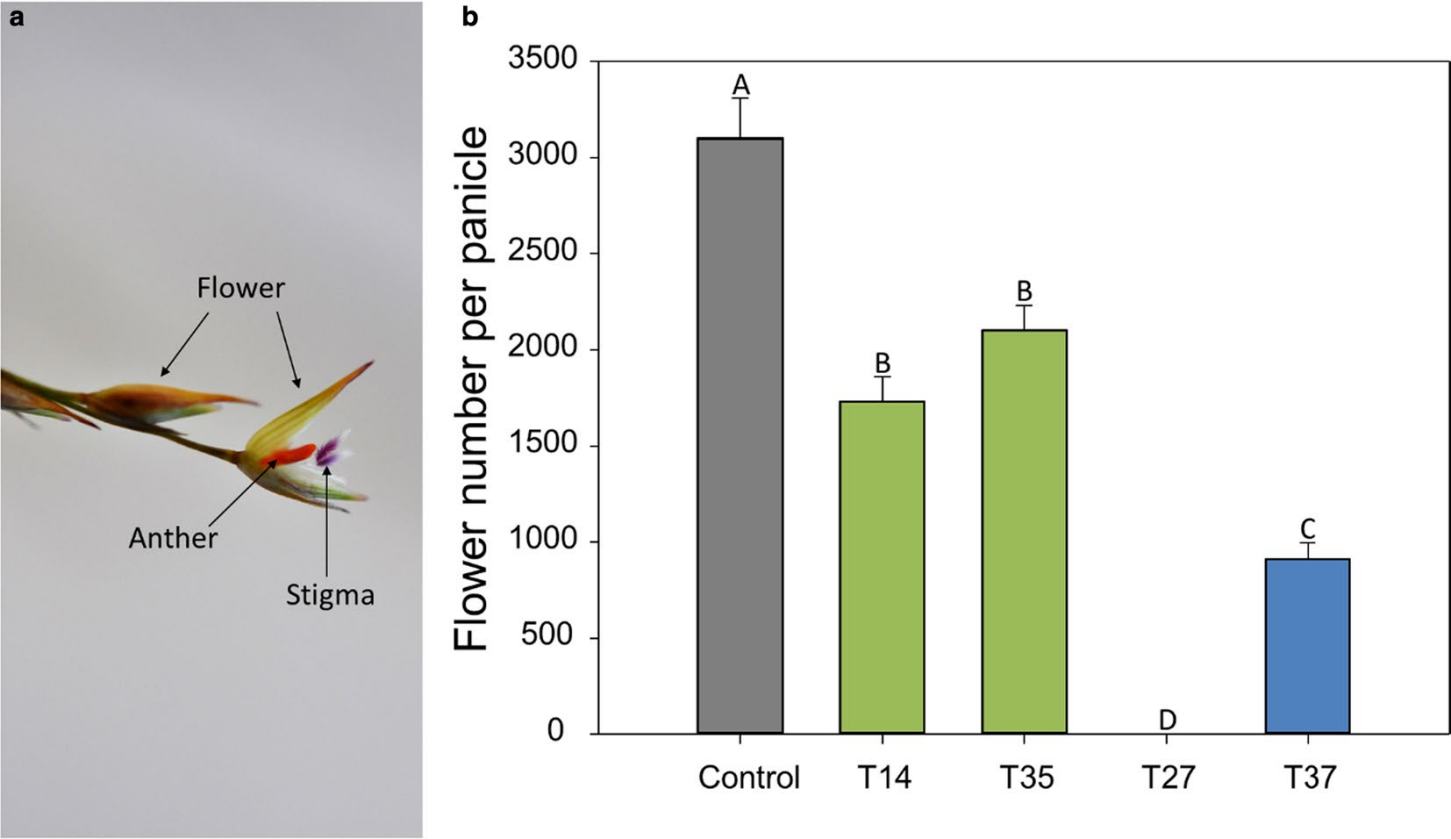
Flower number per panicle in year two (2016). **a** Image of closed and open switchgrass flowers. Taken with a Nikon D90, 60-mm micro lens (Nikon USA, Melville, N.Y.). **b** Letters represent significant differences between means (Fisher’s LSD, *P* < 0.05). Error bars represent standard error of the means. *P* ≤ 0.0001

**Fig. 4:**
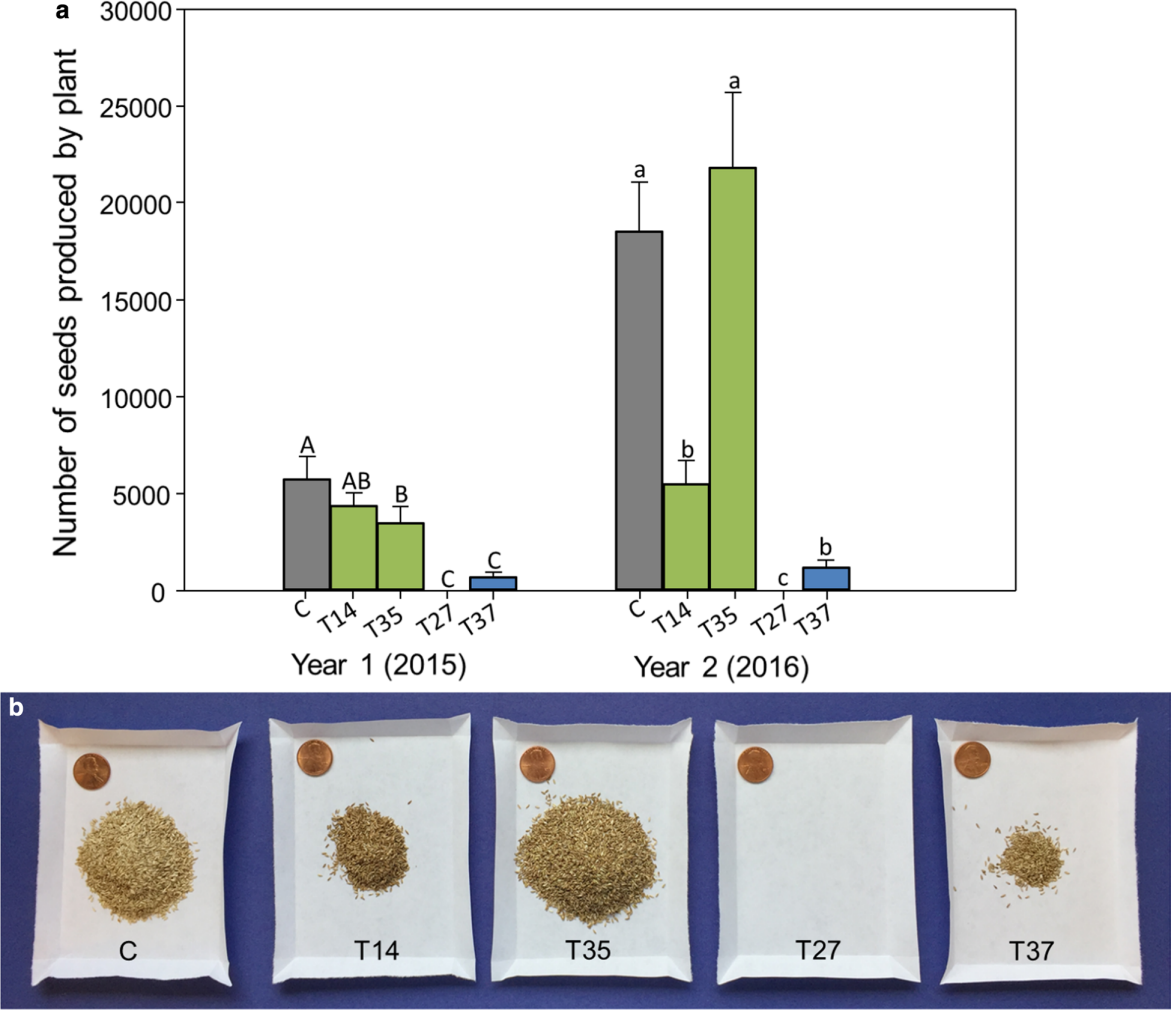
Number of seeds produced by plant for each transgenic line. Lines include the control (C), low miR156 overexpression lines (T14 and T35), and medium miR156 overexpression lines (T27 and T37). **a** Capital letters represent significant differences between means in year one (2015) (*P* ≤ 0.0001), and lowercase letters represent significant differences between means in year two (2016) (*P* ≤ 0.0001; Fisher’s LSD, *P* < 0.05). Error bars represent standard error of the means. **b** Visual representation of the average number of seeds produced per plant in year two (2016). Penny used for scale

The medium overexpression lines (T27 and T37) had notably decreased numbers of flowers that were also produced in a delayed floral transition phase (Figs. 2, 3). Line T27 never produced flowers in the field, but had attenuated biomass production. Only a subset of T37 plants flowered in the field in either growing season. The plants that did flower were delayed 12 weeks after the control in year one and 2 weeks in year two (Fig. 2). T37 panicle number per plant was reduced 65.9% in year one and 23.8% in year two compared to the control, and the panicles were shorter in length (Table 1). The delayed and diminished flowering phenotype led to a commensurate and drastic reduction in both flower and seed production per plant in line T37 compared with the control (Figs. 3, 4). In year one, seed production was reduced 88.2% in T37 plants compared with the control, and in year two seed production was 93.5% less in T37 plants.

**Table 1:** Flowering and reproduction of miR156-overexpressing switchgrass and the nontransgenic control in the field

Year	Line	Panicle number per plant	Panicle length (cm)	Spikelets per panicle	Percent seed germination
2015	C	29.0 ± 1.6^a^	54.33 ± 1.69^a^	N/a	4.75 ± 3.47^b^
	T14	22.2 ± 1.1^b^	49.80 ± 1.29^b^	N/a	5.50 ± 1.89^b^
	T35	22.6 ± 1.7^b^	51.55 ± 1.67^ab^	N/a	22.75 ± 3.97^a^
	T27	0.0 ± 0.0^d^	N/a	N/a	N/a
	T37	9.9 ± 1.7^c^	26.77 ± 2.07^c^	N/a	0.25 ± 0.25^b^
2016	C	103.5 ± 4.0^a^	73.34 ± 0.66^a^	27.5 ± 0.4^a^	34.75 ± 6.30^a^
	T14	60.6 ± 3.0^c^	61.46 ± 0.91^c^	24.6 ± 0.7^c^	15.25 ± 1.93^b^
	T35	98.8 ± 4.7^a^	68.01 ± 0.78^b^	25.8 ± 0.5^bc^	19.25 ± 3.33^b^
	T27	0.0 ± 0.0^d^	N/a	N/a	N/a
	T37	78.9 ± 7.5^b^	40.78 ± 1.26^d^	26.4 ± 1.0^ab^	18.25 ± 2.06^b^

Lines T14 and T35 have low overexpression of miR156, whereas lines T27 and T37 have moderate levels of overexpression of the transgene

Values represent averages ± standard error. Letters indicate significant differences (*P* < 0.05) within year and trait using Fisher’s LSD. Data sets were not compared between years. *N/a* not applicable since there were no flowers produced

All plants in the low overexpression lines flowered both years. T35 flowering phenology was delayed by 6 weeks relative to the control in year one, but was not delayed in year two (Fig. 2). T35 produced 22.1% fewer panicles, but were no different in length than the control (Table 1). The opposite was found in year two; T35 and the control produced the same number of panicles, but T35 panicles were shorter. However, T35 plants produced fewer flowers and seeds than the control for both years (Figs. 3, 4). Line T14 flowered at the same time as the control in year one and two weeks before the control in year two (Fig. 2). Although panicles emerged early in the season, they were fewer and smaller than control panicles (Table 1). T14 also produced fewer flowers and seeds than the control (Figs. 3, 4).

### Seed germination

Seeds from the ‘Alamo’ non-transgenic control and transgenic lines were also collected and germinated. T35 was the only line to differ from the control in year one (18% higher germination), but there were no differences among transgenic lines in year 2, all of which had lower germination frequency than the control (Table 1).

### Aboveground vegetative biomass production and plant morphology

**Fig. 5:**
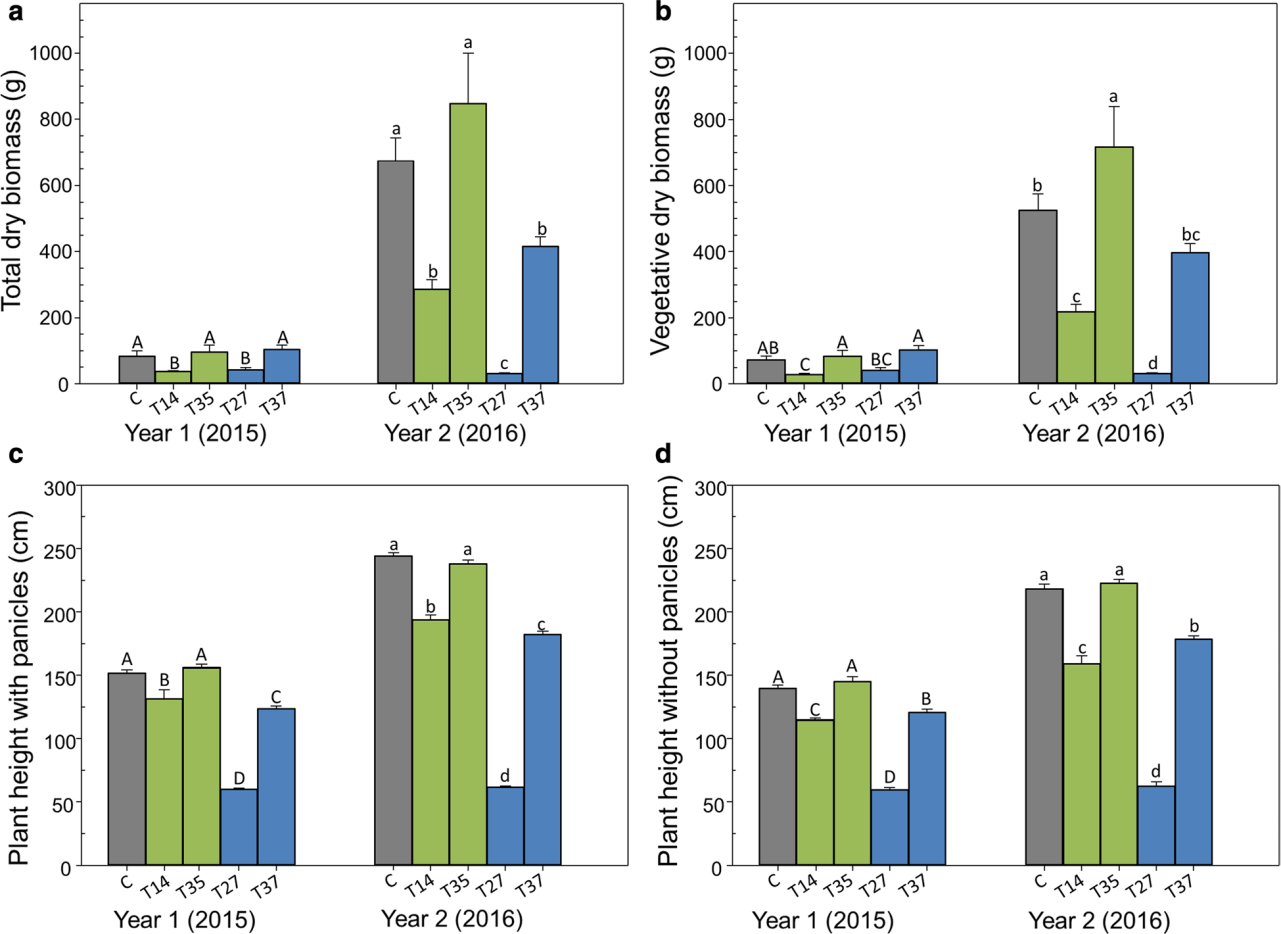
End-of-season dry biomass and height of miR156 transgenic switchgrass and control field grown in East Tennessee for 2 years. Year one growing season took place from June 05 to November 24, 2015. Year two growing season took place from March 30 to November 18, 2016. **a** Dry biomass of both vegetative and reproductive tissues. Year one *P* = 0.0066; year two *P* ≤ 0.0001. **b** Dry biomass without panicles. Year one *P* = 0.002; year two *P* ≤ 0.0001. **c** Tallest part of the plant before panicle removal. *P* ≤ 0.0001 for both years. **d** Plant height after panicle removal. *P* ≤ 0.0001 for both years. Capital letters represent significant differences between means in year one (2015), and lowercase letters represent significant differences between means in year two (2016) (Fisher’s LSD, *P* < 0.05). Error bars represent standard error of the means

Low expressing line T35 most closely resembled the control in the field: these plants had equivalent dry biomass production at the end of both seasons (Fig. 5a), as well as other traits (Table 2; Fig. 5). T35 did produce wider leaves and tillers with a greater stem diameter than the control in year two. Lines T14 and T27 produced less biomass, but line T27 produced the most tillers in year one and was matched only by T37 in year two. T27 plants were shorter (Fig. 5c, d) and with diminutive stem diameters (Table 2), which resulted in very low biomass production (Fig. 5a, b). The biomass of T27 plants was actually reduced by approximately 10 g in the second season (Fig. 5a, b). T14 plants were shorter than the control, and they produced few, slender tillers. Line T37 plants and controls produced equivalent biomass in year one, but the control outperformed T37 in year two (Fig. 5a). However, when panicles are removed from the biomass data, T37 and the control produced statistically equivalent biomass in both years, which is important from a commercialization perspective (Fig. 5b). The difference in plant height is also less drastic when panicles were removed (Fig. 5d). T37 plants had smaller diameter tillers with smaller leaves than the control (Table 2), but the increased tillering of T37 compensated for the stem and leaf traits, contributing to the high biomass production of T37.

**Table 2:** Year one (2015) and year two (2016) end-of-season vegetative morphological data and cell wall characterization of miR156-overexpressing switchgrass and the wild-type control in the field

Year	Line	Tiller number	Leaf length (cm)	Leaf width (cm)	Node number	Internode diameter (mm)	Lignin (% CWR)	S/G ratio	Sugar release (g/g CWR)
2015	C	47.5 ± 3.7^c^	52.5 ± 1.1^a^	1.47 ± 0.02^a^	5.4 ± 0.1^b^	4.73 ± 0.08^a^	20.4 ± 0.4^b^	0.66 ± 0.01^b^	0.47 ± 0.00^ab^
	T14	30.9 ± 2.5^c^	36.6 ± 0.6^c^	1.15 ± 0.02^c^	4.9 ± 0.1^c^	4.25 ± 0.09^b^	21.3 ± 0.2^a^	0.69 ± 0.01^a^	0.44 ± 0.01^b^
	T35	41.2 ± 3.6^c^	48.2 ± 1.3^b^	1.34 ± 0.02^b^	5.1 ± 0.1^bc^	4.95 ± 0.11^a^	21.0 ± 0.1^ab^	0.69 ± 0.01^a^	0.49 ± 0.01^a^
	T27	193.2 ± 11.7^a^	15.3 ± 0.5^e^	0.34 ± 0.01^e^	5.5 ± 0.2^b^	1.27 ± 0.03^d^	20.5 ± 0.3^b^	0.59 ± 0.01^c^	0.44 ± 0.01^b^
	T37	106.0 ± 5.5^b^	27.3 ± 0.9^d^	0.73 ± 0.02^d^	7.8 ± 0.2^a^	3.04 ± 0.06^c^	20.5 ± 0.2^b^	0.59 ± 0.01^c^	0.49 ± 0.03^a^
2016	C	112.2 ± 5.0^b^	52.6 ± 1.1^a^	1.17 ± 0.02^b^	8.0 ± 0.2^c^	5.36 ± 0.07^b^	23.2 ± 0.1^b^	0.66 ± 0.01^ab^	N/a
	T14	66.1 ± 3.7^c^	31.9 ± 1.1^b^	0.86 ± 0.03^c^	7.0 ± 0.2^d^	4.18 ± 0.13^c^	25.0 ± 0.2^a^	0.70 ± 0.02^a^	N/a
	T35	108.6 ± 6.8^b^	48.5 ± 0.8^a^	1.26 ± 0.03^a^	7.7 ± 0.1 ^cd^	5.79 ± 0.07^a^	23.1 ± 0.1^b^	0.64 ± 0.03^b^	N/a
	T27	172.7 ± 16.0^a^	9.6 ± 0.4^c^	0.22 ± 0.01^e^	8.8 ± 0.3^b^	0.99 ± 0.03^e^	21.4 ± 0.4^c^	0.57 ± 0.00^c^	N/a
	T37	182.2 ± 6.6^a^	29.8 ± 1.1^b^	0.58 ± 0.02^d^	11.1 ± 0.2^a^	3.37 ± 0.06^d^	22.8 ± 0.2^b^	0.58 ± 0.01^c^	N/a

Values represent averages ± standard error. Letters indicate significant differences (*P* < 0.05) within year and trait using Fisher’s LSD. Data sets were not compared between years

*CWR* cell wall residue, *S/G* syringyl/guaiacyl

Cell wall composition (lignin content, digestibility, and sugar release) of the transgenic switchgrass lines had a few notable changes compared with the control. In both seasons, line T14 plant cells contained more lignin than the control (Table 2). T14, along with line T35 (both low overexpression lines), had higher S/G ratios than the control which suggests that they are more easily digestible (Table 2) [46]. Both medium overexpression lines (T27 and T37) had lower S/G ratios than the control in both seasons. Transgenic lines did not differ from the control in sugar release (Table 2).

### Transcriptomic analysis

**Fig. 6:**
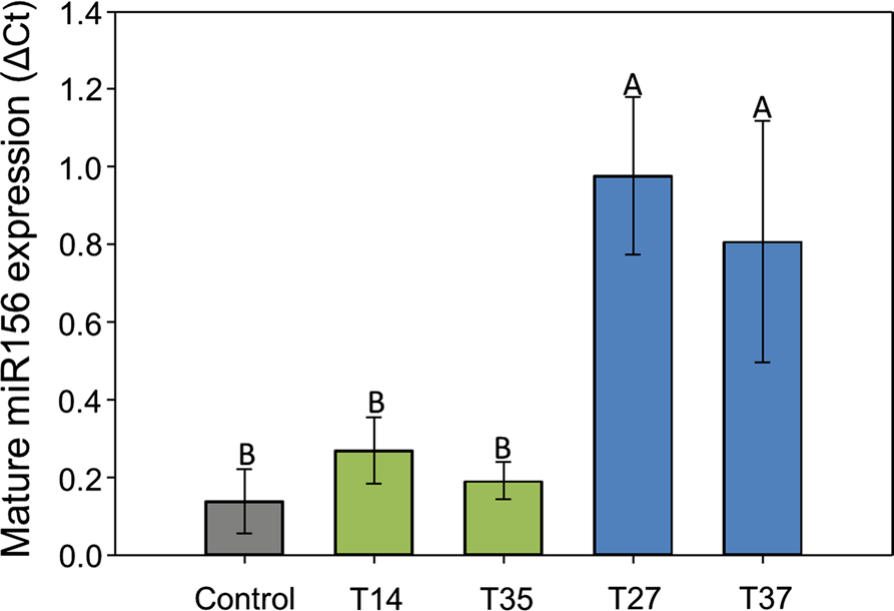
Relative mature miR156 expression results from qRT-PCR. The expression level of miR156 was normalized using miR390 expression. Combined leaf and tiller tissue from V3 stage tillers harvested in year 2 (2016) was used for mRNA extraction. Letters represent significant differences between means (Fisher’s LSD, *P* < 0.05). Error bars represent standard error of the means. *P* = 0.0103

The level of mature miR156 transcript was examined using quantitative RT-PCR, and results were congruent with the results of the same clonal lines grown under greenhouse conditions [36] and in the field in which panicle removal was required [37]. Lines categorized as low overexpressors (T14 and T35) had three and two times increase, respectively, in miR156 levels compared to control plants in the field. Medium overexpression lines (T27 and T37) show 10 and eight times increase, respectively, in mature miR156 levels compared to the control (Fig. 6).

The expression levels of four *SPL* genes (*PvSPL1, PvSPL2, PvSPL3,* and *PvSPL6*) were also examined using quantitative RT-PCR to determine the effects of miR156 overexpression on its target genes in field-grown plants. All expression levels were examined on V3 stage vegetative tillers collected in year two (2016). The high variation among biological replicates resulted in no statistically significant differences for the expression levels of any *SPL* genes. Line T27 had the highest miR156 expression and showed the lowest *PvSPL* expression in general (Additional file 1: Figure S3).

The V3 stage tillers collected from the field in year one (2015) represent mid-season aboveground biomass for the global transcriptomic analysis (microarrays). Total RNA from all four transgenic lines and the ‘Alamo’ wild-type control was analyzed using Affymetrix microarray chips. Of the 85,587 probe sets examined, 14,507 were significantly up- or downregulated for one or more of the transgenic lines. Genes related to the miR156 pathway and flowering were chosen for further examination. Of 49 probe sets annotated as *SPL* according to known *Arabidopsis thaliana* and rice *SPL* sequences, eight *SPL* probes were found to be downregulated in open-flowering field conditions (Additional file 2: Table S1). *SPL* downregulation was negatively associated with mature miR156 overexpression (Fig. 6, Additional file 1: Figure S3). For the highest miR156 overexpression line T27, all eight *SPL* gene annotations were significantly downregulated (Additional file 2: Table S1). Six *SPL* gene annotations were downregulated in T37, which had the second highest miR156 overexpression. The expression of *SPL* genes appeared to have similar patterns to nontransgenics in the low overexpression lines; only two *SPL* genes were downregulated in T14, and none were downregulated in T35 (Additional file 2: Table S1). Probes corresponding to other important genes involved in flowering pathways, such as Arabidopsis *AtFT* (*Flowering Locus T*)/rice *OsFTL* (*Flowering Locus T*-*Like*) genes, were also significantly affected in miR156-overexpressing switchgrass (Additional file 2: Table S1).

## Discussion

Exploiting gene regulation by manipulating miRNAs could be useful in the sustained use of genetically engineered biofuel feedstocks to enhance desired traits such as abiotic and biotic stress responses, biomass yield, and lignin content [24–30]. miR156 targets the SQUAMOSA PROMOTER BINDING PROTEIN-LIKE (*SPL*) transcription factor family, which is involved in many plant developmental processes including the vegetative to reproductive phase developmental transition [32–35, 47]. The overexpression of miR156 has been shown to delay flowering and increase biomass yield in multiple plant species [36, 48–51]. *Arabidopsis thaliana* plants engineered to overexpress miR156 had a moderate delay in flowering and an increase in total leaf number when grown under long days [48]. A similar phenotype was seen in red clover (*Trifolium pratense* L.) engineered to overexpress miR156; transgenic red clover plants had an increased number of shoots and delayed flowering [51]. Some transgenic events of switchgrass engineered to overexpress maize *Corngrass1*, a gene in the miR156 class of miRNAs, did not flower in a one-season California field trial, and weak overexpression levels did not affect biomass production [50]. Transgenic switchgrass that overexpressed a rice miR156 precursor produced no flowering lines when grown in the greenhouse, and the low and medium overexpression lines produced more biomass than the control [36].

### *SPL* downregulation causes delayed flowering in the field

Latitudinal origin and divergence of traits such as flowering time, growth and phenotype architecture, and disease susceptibility are used to classify switchgrass into either upland or lowland ecotypes [52–60]. Lowland switchgrass typically flowers later than varieties that originated in the north due to an elongated growth period [60]. ‘Alamo,’ a lowland ecotype of switchgrass, typically flowers in mid–late June when grown in the southern United States [54]. This study observed non-transgenic ‘Alamo’ switchgrass panicle production in mid- to late June for both growing seasons. Because the ‘Alamo’ nontransgenic control flowered in the same period as in the past studies [54, 60], a delayed flowering phenotype observed in transgenic lines can be contributed to miR156 overexpression rather than environmental effects. Transgenic lines T14, T35, and T37 flowered in the field. While this phenotype was different than the previous greenhouse study [36], the same was reported in a field study in Knoxville, Tenn. using the same miR156-overexpressing plants [37]. Over the course of 3 years, T27 was the only line that did not produce panicles [37]. *SPL3* is an important upstream activator of floral meristem identity genes such as *LEAFY*, *FRUITFULL*, and *APETALA1* [61], and the microarray revealed significant downregulation of *SPL3* and *APETALA1* in lines T27 and T37 (Additional file 2: Table S1). The medium overexpression lines were the only transgenic lines to have significant downregulation in *SPL3, SPL4,* and *SPL5*, which have overlapping functions to promote floral induction and transform the vegetative meristem to an inflorescence meristem [62, 63]. This downregulation of important *SPL* genes explains the delayed and non-flowering phenotypes of these two transgenic lines.

We observed that all transgenic lines produced shorter panicles than the control in year two, and lines T14 and T37 were also shorter in year one (Table 1). Overexpression of miR156 in rice resulted in short panicles with reduced spikelet and grain number [64]. Line T37 was the only transgenic line to consistently produce fewer panicles and seeds than the control. While Xie et al. [64] found no difference in seed fertility, all miR156 switchgrass transgenic lines had lower seed germination than the control in year two (Table 1).

### *SPL* downregulation results in altered plant phenotype

The trend in overexpression of miR156 in field-grown plants was consistent with that of previous greenhouse and field studies, as was the inverse relationship between miR156 and *SPL* gene target abundance (Fig. 6, Additional file 1: Figure S1) [36, 37]. Medium overexpression lines (T27 and T37) produced a high number of tillers, which is a common occurrence in plants overexpressing miR156 [36, 37, 48, 50, 51]. The high tiller number and short stature of T27 are most likely caused by a reduction in *SPL1* and *SPL2* expression (Additional file 1: Figure S4, Additional file 2: Table S1) which are important for tiller initiation and internode elongation [65]. T27 and T37 tillers were thin compared to the control, and the leaves were smaller in both length and width for both lines (Table 2). When *Arabidopsis thaliana* was engineered to constitutively express miR156, plants produced leaves that resembled juvenile leaves in size, shape, and trichome production [34]. miR156 promotes the expression of juvenile leaf traits by repressing *SPL* genes involved in plant maturation, such as *SPL2/10* and *SPL3/4/5*, all of which were reduced in T27 and T37 (Additional file 2: Table S1) [34, 49, 62, 63, 65, 66]. The observed trends in vegetative biomass, height (without panicles), and tiller number were similar in ranking for year two data between this study and Baxter et al. [37], even though the latter study required panicle removal as a federal regulatory requirement in the field release permit. The high tiller number of line T37 without a reduction in height ‘rescued’ its biomass production. A miR156 overexpression level that falls between that of line T27 and line T37 (Fig. 6) would be ideal as it would most likely result in a non-flowering, high-yielding line. Such expression may be, ideally, triggered by environmental or developmental cues.

## Conclusions

This two-year field study of miR156-overexpressing transgenic switchgrass is the first field experiment in the eastern U.S. in which USDA-APHIS-BRS regulators allowed open flowering. Thus, the present study was the first opportunity to closely examine the dynamics of switchgrass reproduction in the field using transgenic lines with a range of a miR156 expression. We found that medium overexpression levels of miR156 such as those in line T37 resulted in delayed and reduced flowering accompanied by high biomass production. Panicle size, seed production, and seed germination were also significantly reduced compared to the control. This outcome is the result of the downregulation of important miR156 *SPL* gene targets including *SPL2/10* and *SPL3/4/5*. If miR156 overexpression was tied to developmental or environmental cues via conditional expression, then it could further optimize the use of miR156 overexpression as a bioconfinement tool.

## Additional files

**Additional file 1.** Supplemental figures.

**Additional file 2.**  Supplemental table.
