# Supplementary material for: Field-grown miR156 transgenic switchgrass reproduction, yield, global gene expression analysis, and bioconfinement
Source: Biotechnol Biofuels. 2017 Nov 30;10:255. doi: 10.1186/s13068-017-0939-1 (PMC5707911; doi:10.1186/s13068-017-0939-1)
Supplement: Supplementary file 1 — Additional file 1. Supplemental figures. [file 13068_2017_939_MOESM1_ESM.docx]

Additional File 1

Supplemental Figures


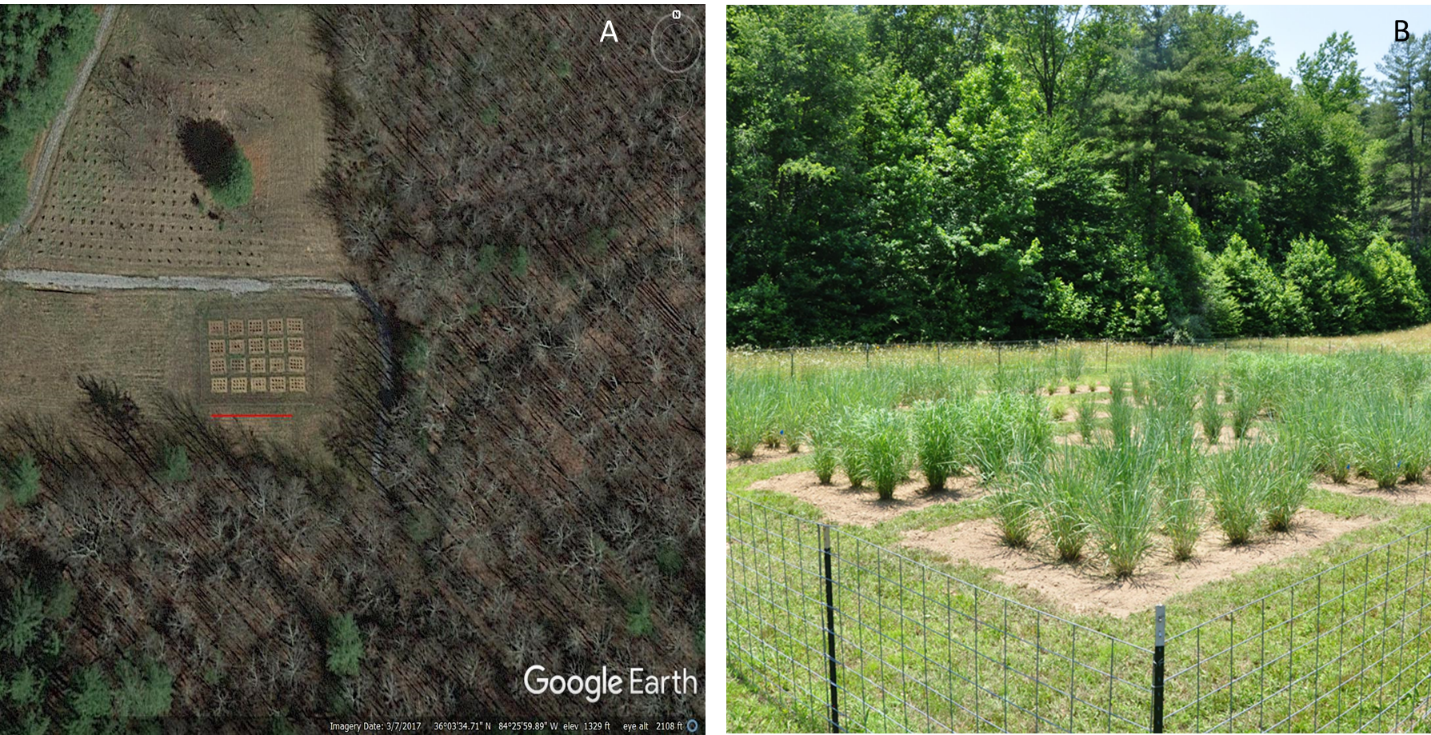


**Figure S1.** Images of the field site located in Oliver Springs, Tennessee, USA. A) Google Earth image showing the heavily forested border of the field. Satellite image was taken on March 07, 2017. The red line represents a 20 m length. B) View of the experimental plots from the NE corner facing SW during year two on June 14, 2016.


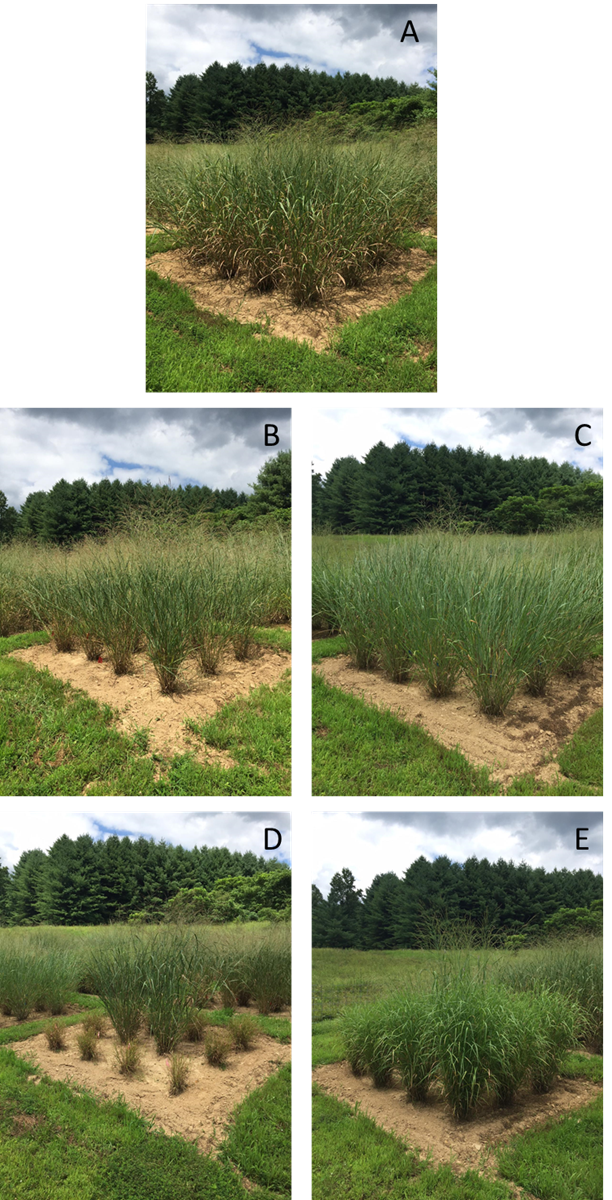


**Figure S2.** Representative images of each line in the field. Pictures were taken in year two on August 11, 2016. A) Non-transgenic ‘Alamo.’ B) Low overexpression line T14. C) Low overexpression line T35. D) Medium overexpression line T27 surrounding easily visible ST2 non-transgenic plants in the center of the plot. E) Medium overexpression line T37.


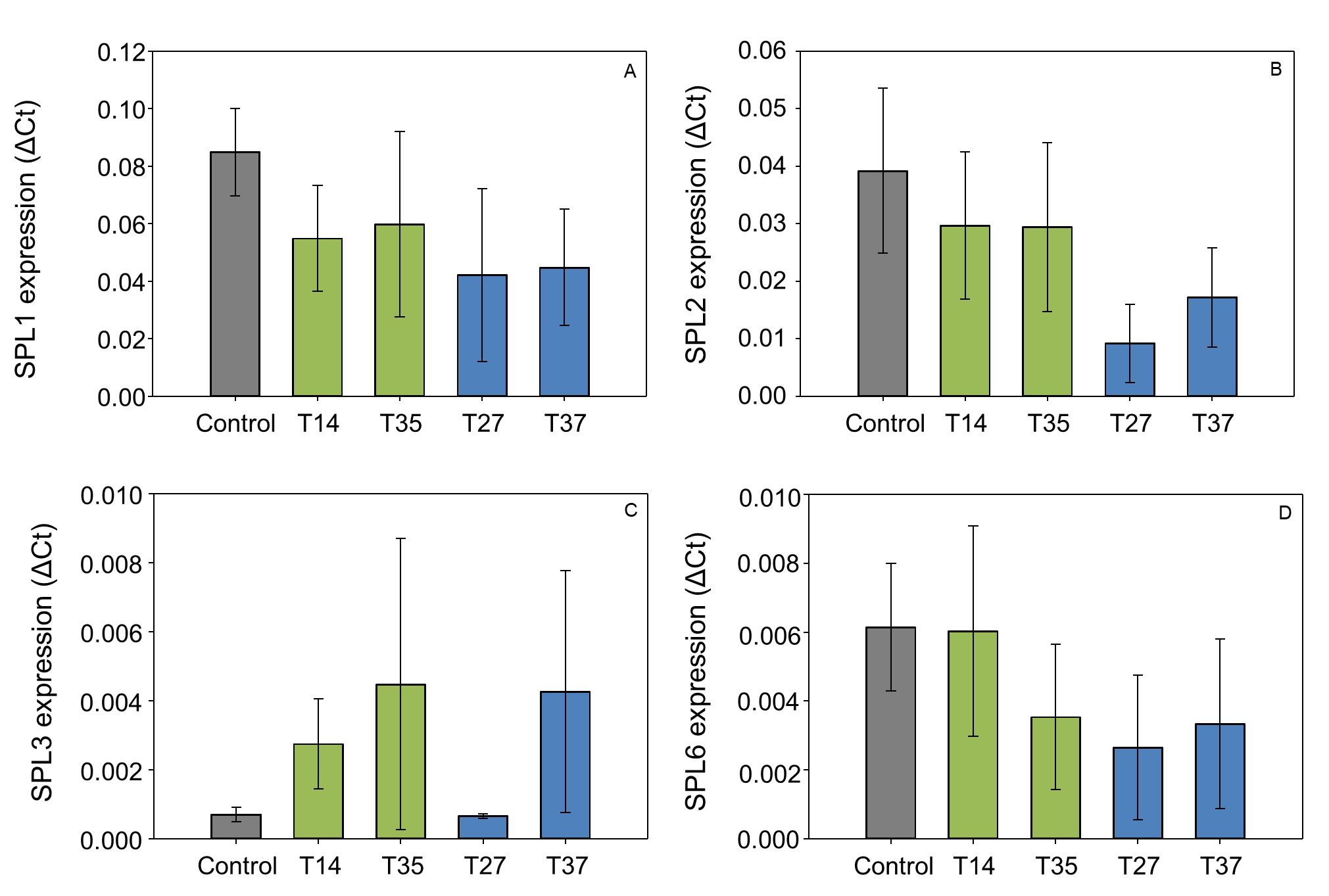


**Figure S3.** qRT-PCR results of combined V3 tiller and leaf tissue by plot using the PvSPL primers. Error bars represent ± standard error of the means. No significant differences were found between means of any of the four target SPL genes. A) SPL1 expression (P = 0.7374; ANOVA). B) SPL2 expression (P = 0.4402; ANOVA). C) SPL3 expression (P = 0.8544). D) SPL6 expression (P = 0.7508; ANOVA).
