# Supplementary material for: Field-grown miR156 transgenic switchgrass reproduction, yield, global gene expression analysis, and bioconfinement
Source: Biotechnol Biofuels. 2017 Nov 30;10:255. doi: 10.1186/s13068-017-0939-1 (PMC5707911; doi:10.1186/s13068-017-0939-1)
Supplement: Supplementary file 2 — Additional file 2. Supplemental table. [file 13068_2017_939_MOESM2_ESM.docx]

**Supplemental File 2**

**Table S1**. Summary of transcriptomic analysis of relative expression of transgenic lines using gene-specific Affymetrix microarray genechips. The values represent the normalized fraction of expression relative to the nontransgenic value of each gene; transgenic/control (Ctrl). SPL, Squamosa Promotor Binding Protein-Like; FT, Flowering Locus T; FTL, Flowering Locus T-Like; FPF, Flowering Promoting Factor; FPFL, Flowering Promoting Factor-Like; PFT, Phytochrome and Flowering Time regulatory protein; AP, Apetala; MADS, MADS-box genes; AGL, Agamous-Like; SEP, Sepallata; LEA, Late Embryogenesis Abundant; SAG, Senescence-Associated Genes; PI, Pistillata; AG, Agamous; STK, Seedstick; SVP, Short Vegetative Phase; DIA, AGL61/Diana; ERD, Early Responsive to Dehydration; SOC, Suppressor of Overexpression of Constans; COL, Constans-Like

| **Probe set** | **Annotation** | **Short name** | **T-14/Ctrl** | **T-35/Ctrl** | **T-27/Ctrl** | **T-37/Ctrl** |
| --- | --- | --- | --- | --- | --- | --- |
| Pavir.2NG503700.1_at | Best-hit-arabi-name=AT3G15270.1 / Best-hit-rice-name=LOC_Os07g32170.1 | AtSPL5 / OsSPL13 | 0.36 | - | 0.24 | - |
| Pavir.2NG503500.1_at | Best-hit-arabi-name=AT1G53160.1 / Best-hit-rice-name=LOC_Os07g32170.1 | AtSPL4 / OsSPL13 | 0.41 | - | 0.24 | 0.43 |
| Pavir.2KG430000.1_at | Best-hit-arabi-name=AT2G33810.1 / Best-hit-rice-name=LOC_Os07g32170.1 | AtSPL3 / OsSPL13 | - | - | 0.21 | 0.48 |
| Pavir.2KG430400.1_at | Best-hit-arabi-name=AT3G15270.1 / Best-hit-rice-name=LOC_Os07g32170.1 | AtSPL5 / OsSPL13 | - | - | 0.21 | 0.33 |
| Pavir.1NG028400.1_x_at | Best-hit-arabi-name=AT1G27370.3 / Best-hit-rice-name=LOC_Os02g04680.2 | AtSPL10 / OsSPL3 | - | - | 0.31 | 0.37 |
| Pavir.1NG028400.2_x_at | Best-hit-arabi-name=AT1G27370.3 / Best-hit-rice-name=LOC_Os02g04680.2 | AtSPL10 / OsSPL3 | - | - | 0.31 | 0.37 |
| Pavir.1KG076500.1_at | Best-hit-arabi-name=AT5G43270.3 / Best-hit-rice-name=LOC_Os06g45310.1 | AtSPL2 / OsSPL11 | - | - | 0.38 | - |
| Pavir.1KG043600.1_at | Best-hit-arabi-name=AT1G27370.3 / Best-hit-rice-name=LOC_Os02g04680.2 | AtSPL10 / OsSPL3 | - | - | 0.29 | 0.41 |
| Pavir.5NG100600.1_at | Best-hit-arabi-name=AT1G65480.1 / Best-hit-rice-name=LOC_Os01g11940.1 | AtFT / OsFTL1 | 0.45 | - | 0.14 | - |
| Pavir.5NG198400.1_x_at | Best-hit-arabi-name=AT1G65480.1 / Best-hit-rice-name=LOC_Os05g44180.1 | AtFT / OsFTL10 | 0.48 | - | 0.41 | 2.43 |
| Pavir.3KG344200.1_x_at | Best-hit-arabi-name=AT1G65480.1 / Best-hit-rice-name=LOC_Os05g44180.1 | AtFT / OsFTL10 | - | - | - | 4.70 |
| Pavir.3KG349500.1_x_at | Best-hit-arabi-name=AT1G65480.1 / Best-hit-rice-name=LOC_Os05g44180.1 | AtFT / OsFTL10 | - | - | - | 4.70 |
| Pavir.5KG284600.1_s_at | Best-hit-arabi-name=AT1G65480.1 / Best-hit-rice-name=LOC_Os06g35940.1 | AtFT / OsFTL12 | - | - | 0.44 | - |
| Pavir.4KG264600.1_at | Best-hit-arabi-name=AT1G65480.1 / Best-hit-rice-name=LOC_Os06g35940.1 | AtFT / OsFTL12 | - | - | 0.43 | - |
| Pavir.5KG751900.1_at | Best-hit-arabi-name=AT5G24860.1 / Best-hit-rice-name=LOC_Os01g70730.1 | AtFPF1 / OsFPFL1 | - | 0.50 | - | - |
| Pavir.4KG047800.1_at | Best-hit-arabi-name=AT1G65480.1 / Best-hit-rice-name=LOC_Os06g06320.1 | AtFT / OsFTL2 | - | - | 0.20 | - |
| Pavir.J024900.1_at | Best-hit-arabi-name=AT5G10625.1 / Best-hit-rice-name=LOC_Os02g26210.1 | AtFPF1 / OsFPFL1 | - | - | 0.46 | - |
| Pavir.5KG166700.1_at | Best-hit-arabi-name=AT5G24860.1 / Best-hit-rice-name=LOC_Os01g15340.1 | AtFPF1 / OsFPFL1 | - | - | 2.01 | - |
| Pavir.5KG029000.1_at | Best-hit-arabi-name=AT1G65480.1 / Best-hit-rice-name=LOC_Os01g11940.1 | AtFT / OsFTL1 | - | - | 0.36 | - |
| Pavir.2KG594700.1_x_at | Best-hit-arabi-name=AT5G24860.1 / Best-hit-rice-name=LOC_Os07g47450.1 | AtFPF1 / OsFPFL1 | - | - | - | 2.78 |
| Pavir.2NG627900.1_at | Best-hit-arabi-name=AT5G24860.1 / Best-hit-rice-name=LOC_Os07g47450.1 | AtFPF1 / OsFPFL1 | - | 2.52 | - | 3.60 |
| Pavir.J167400.1_s_at | Best-hit-arabi-name=AT1G25540.2 / Best-hit-rice-name=LOC_Os09g13610.1 | AtPFT1 / OsPFT1 | 2.15 | - | - | - |
| Pavir.J167400.2_s_at | Best-hit-arabi-name=AT1G25540.2 / Best-hit-rice-name=LOC_Os09g13610.1 | AtPFT1 / OsPFT1 | 2.15 | - | - | - |
| Pavir.4NG331800.1_at | Best-hit-arabi-name=AT3G54340.1 / Best-hit-rice-name=LOC_Os06g49840.1 | AtAP3 / OsMADS16 | 0.11 | - | 0.01 | - |
| Pavir.6KG379800.1_x_at | Best-hit-arabi-name=AT1G24260.1 / Best-hit-rice-name=LOC_Os08g41950.2 | AtAGL9, AtSEP3 / OsMADS7 | 0.14 |  | 0.06 |  |
| Pavir.6NG327400.1_at | Best-hit-arabi-name=AT1G24260.1 / Best-hit-rice-name=LOC_Os08g41950.2 | AtAGL9, AtSEP3 / OsMADS7 | 0.19 | - | 0.04 | - |
| Pavir.1KG449500.1_at | Best-hit-arabi-name=AT2G45650.1 / Best-hit-rice-name=LOC_Os02g45770.1 | AtAGL6 / OsMADS6 | 0.19 | - | 0.05 | 0.43 |
| Pavir.2NG422200.1_at | Best-hit-arabi-name=AT1G24260.1 / Best-hit-rice-name=LOC_Os09g32948.1 | AtAGL9, AtSEP3 / OsMADS8 | 0.20 | - | 0.03 | - |
| Pavir.2NG419400.2_s_at | Best-hit-arabi-name=AT1G24260.2 / Best-hit-rice-name=LOC_Os09g32948.1 | AtAGL9, AtSEP3 / OsMADS8 | 0.22 | - | 0.02 | - |
| Pavir.2NG419400.3_s_at | Best-hit-arabi-name=AT1G24260.2 / Best-hit-rice-name=LOC_Os09g32948.1 | AtAGL9, AtSEP3 / OsMADS8 | 0.22 | - | 0.02 | - |
| Pavir.5NG221200.1_at | Best-hit-arabi-name=AT4G02380.1 / Best-hit-rice-name=LOC_Os01g21250.1 | AtLEA5, AtSAG21 | 0.23 | 0.26 | - | - |
| Pavir.1NG424900.2_s_at | Best-hit-arabi-name=AT2G45650.1 / Best-hit-rice-name=LOC_Os02g45770.1 | AtAGL6 / OsMADS6 | 0.24 | - | 0.10 | - |
| Pavir.1NG424900.1_x_at | Best-hit-arabi-name=AT2G45650.1 / Best-hit-rice-name=LOC_Os02g45770.1 | AtAGL6 / OsMADS6 | 0.27 | - | 0.12 | - |
| Pavir.1NG424900.3_x_at | Best-hit-arabi-name=AT2G45650.1 / Best-hit-rice-name=LOC_Os02g45770.1 | AtAGL6 / OsMADS6 | 0.27 | - | 0.12 | - |
| Pavir.J149100.1_s_at | Best-hit-arabi-name=AT5G20240.1 / Best-hit-rice-name=LOC_Os05g34940.2 | AtPI / OsMADS4 | 0.27 | - | 0.10 | - |
| Pavir.9NG641900.1_at | Best-hit-arabi-name=AT3G02310.1 / Best-hit-rice-name=LOC_Os03g11614.1 | AtAGL4, AtSEP2 / OsMADS1 | 0.28 | - | 0.20 | - |
| Pavir.J149100.2_x_at | Best-hit-arabi-name=AT5G20240.1 / Best-hit-rice-name=LOC_Os05g34940.3 | AtPI / OsMADS4 | 0.28 | - | 0.10 | - |
| Pavir.J149100.3_x_at | Best-hit-arabi-name=AT5G20240.1 / Best-hit-rice-name=LOC_Os05g34940.2 | AtPI / OsMADS4 | 0.28 | - | 0.10 | - |
| Pavir.5NG045000.1_s_at | Best-hit-arabi-name=AT4G18960.1 / Best-hit-rice-name=LOC_Os01g10504.3 | AtAG / OsMADS3 | - | - | 0.03 | - |
| Pavir.5NG045000.2_s_at | Best-hit-arabi-name=AT4G18960.1 / Best-hit-rice-name=LOC_Os01g10504.2 | AtAG / OsMADS3 | - | - | 0.03 | - |
| Pavir.5NG045000.3_x_at | Best-hit-arabi-name=AT4G18960.1 / Best-hit-rice-name=LOC_Os01g10504.1 | AtAG / OsMADS3 | - | - | 0.04 | - |
| Pavir.5NG045000.4_x_at | Best-hit-arabi-name=AT4G18960.1 / Best-hit-rice-name=LOC_Os01g10504.1 | AtAG / OsMADS3 | - | - | 0.04 | - |
| Pavir.5NG045000.5_x_at | Best-hit-arabi-name=AT4G18960.1 / Best-hit-rice-name=LOC_Os01g10504.1 | AtAG / OsMADS3 | - | - | 0.04 | - |
| Pavir.5KG667500.1_s_at | Best-hit-arabi-name=AT5G20240.1 / Best-hit-rice-name=LOC_Os01g66030.1 | AtPI / OsMADS2 | - | - | 0.09 | - |
| Pavir.5KG676500.1_s_at | Best-hit-arabi-name=AT5G20240.1 / Best-hit-rice-name=LOC_Os01g66030.1 | AtPI / OsMADS2 | - | - | 0.09 | - |
| Pavir.4NG059400.1_s_at | Best-hit-arabi-name=AT1G24260.2 / Best-hit-rice-name=LOC_Os06g06750.1 | AtAGL9, AtSEP3 / OsMADS5 | 0.36 | - | 0.17 | 0.44 |
| Pavir.4NG059400.2_s_at | Best-hit-arabi-name=AT1G24260.1 / Best-hit-rice-name=LOC_Os06g06750.1 | AtAGL9, AtSEP3 / OsMADS5 | 0.36 | - | 0.17 | 0.44 |
| Pavir.9NG415300.1_at | Best-hit-arabi-name=AT3G54340.1 / Best-hit-rice-name=LOC_Os06g49840.2 | AtAP3 / OsMADS16 | 0.37 | - | 0.04 | - |
| Pavir.9KG622200.1_at | Best-hit-arabi-name=AT3G02310.1 / Best-hit-rice-name=LOC_Os03g11614.1 | AtAGL4, AtSEP2 / OsMADS1 | 0.43 | - | 0.45 | - |
| Pavir.4KG066600.1_s_at | Best-hit-arabi-name=AT1G24260.1 / Best-hit-rice-name=LOC_Os06g06750.1 | AtAGL9, AtSEP3 / OsMADS5 | 0.43 | - | 0.17 | 0.38 |
| Pavir.8KG297500.1_at | Best-hit-arabi-name=AT1G71190.1 / Best-hit-rice-name=LOC_Os01g73120.1 | AtSAG18 | 0.44 | 0.13 | 0.22 | 0.35 |
| Pavir.5NG475300.1_x_at | Best-hit-arabi-name=AT4G09960.2 / Best-hit-rice-name=LOC_Os01g52680.1 | AtAGL11, AtSTK / OsMADS32 | 0.45 | - | 0.46 | - |
| Pavir.3KG063200.1_at | Best-hit-arabi-name=AT4G18960.1 / Best-hit-rice-name=LOC_Os12g10540.1 | AtAG / OsMADS13 | - | - | 0.32 | - |
| Pavir.J610400.1_x_at | Best-hit-arabi-name=AT4G18960.1 / Best-hit-rice-name=LOC_Os05g11414.1 | AtAG / OsMADS58 | - | - | 0.35 | - |
| Pavir.3KG091900.1_x_at | Best-hit-arabi-name=AT4G18960.1 / Best-hit-rice-name=LOC_Os05g11414.1 | AtAG / OsMADS58 | - | - | 0.42 | - |
| Pavir.3KG091900.2_x_at | Best-hit-arabi-name=AT4G18960.1 / Best-hit-rice-name=LOC_Os05g11414.1 | AtAG / OsMADS58 | - | - | 0.42 | - |
| Pavir.3KG523200.1_s_at | Best-hit-arabi-name=AT3G02310.1 / Best-hit-rice-name=LOC_Os03g54170.1 | AtAGL4, AtSEP2 / OsMADS34 | - | - | 0.34 | 0.43 |
| Pavir.3KG523200.2_s_at | Best-hit-arabi-name=AT3G02310.1 / Best-hit-rice-name=LOC_Os03g54170.1 | AtAGL4,SEP2 / OsMADS34 | - | - | 0.34 | 0.43 |
| Pavir.5KG517700.1_x_at | Best-hit-arabi-name=AT4G09960.2 / Best-hit-rice-name=LOC_Os01g52680.1 | AtAGL11, AtSTK / OsMADS32 | - | 2.54 | - | - |
| Pavir.5KG517700.2_x_at | Best-hit-arabi-name=AT4G09960.2 / Best-hit-rice-name=LOC_Os01g52680.1 | AtAGL11, AtSTK / OsMADS32 | - | 2.54 | - | - |
| Pavir.5KG518600.1_s_at | Best-hit-rice-name=LOC_Os01g52680.1 | OsMADS32 | - | 2.76 | - | - |
| Pavir.2KG220600.2_at | Best-hit-arabi-name=AT5G45890.1 / Best-hit-rice-name=LOC_Os09g38920.1 | AtSAG12 | - | - | 2.23 | - |
| Pavir.5KG736600.3_at | Best-hit-arabi-name=AT4G11880.1 / Best-hit-rice-name=LOC_Os01g69850.1 | AtAGL14 / OsMADS65 | - | 2.51 | - | - |
| Pavir.4NG131700.1_at | Best-hit-arabi-name=AT2G22540.1 / Best-hit-rice-name=LOC_Os02g52340.1 | AtAGL22, AtSVP / OsMADS22 | - | - | 0.39 | - |
| Pavir.5KG518600.2_x_at | Best-hit-rice-name=LOC_Os01g52680.1 | OsMADS32 | - | 3.35 | - | - |
| Pavir.1NG073400.1_at | Best-hit-arabi-name=AT5G24930.1 / Best-hit-rice-name=LOC_Os02g08150.1 | AtCOL4 | - | - | 0.18 | - |
| Pavir.5KG325100.1_at | Best-hit-arabi-name=AT4G02380.1 / Best-hit-rice-name=LOC_Os05g29930.1 | AtLEA5, AtSAG21 | - | 5.05 | - | 3.17 |
| Pavir.2KG531900.1_at | Best-hit-arabi-name=AT1G69120.1 / Best-hit-rice-name=LOC_Os07g41370.1 | AtAGL7, AtAP1 / OsMADS18 | - | 0.43 | - | - |
| Pavir.3KG523400.1_x_at | Best-hit-arabi-name=AT1G69120.1 / Best-hit-rice-name=LOC_Os03g54160.2 | AtAGL7, AtAP1 / OsMADS14 | - | - | 0.21 | - |
| Pavir.3KG523400.2_x_at | Best-hit-arabi-name=AT1G69120.1 / Best-hit-rice-name=LOC_Os03g54160.2 | AtAGL7, AtAP1 / OsMADS14 | - | - | 0.21 | - |
| Pavir.J371200.1_x_at | Best-hit-arabi-name=AT1G69120.1 / Best-hit-rice-name=LOC_Os03g54160.2 | AtAGL7, AtAP1 / OsMADS14 | - | - | 0.24 | - |
| Pavir.9NG097300.1_x_at | Best-hit-arabi-name=AT1G69120.1 / Best-hit-rice-name=LOC_Os03g54160.2 | AtAGL7, AtAP1 / OsMADS14 | - | - | 0.17 | - |
| Pavir.9NG097300.2_x_at | Best-hit-arabi-name=AT1G69120.1 / Best-hit-rice-name=LOC_Os03g54160.2 | AtAGL7, AtAP1 / OsMADS14 | - | - | 0.17 | - |
| Pavir.2KG001200.1_s_at | Best-hit-arabi-name=AT1G69120.1 / Best-hit-rice-name=LOC_Os07g01820.3 | AtAGL7, AtAP1 / OsMADS15 | - | - | 0.13 | - |
| Pavir.2KG001200.2_s_at | Best-hit-arabi-name=AT1G69120.1 / Best-hit-rice-name=LOC_Os07g01820.3 | AtAGL7, AtAP1 / OsMADS15 | - | - | 0.13 | - |
| Pavir.2NG003000.1_at | Best-hit-arabi-name=AT1G69120.1 / Best-hit-rice-name=LOC_Os07g01820.3 | AtAGL7, AtAP1 / OsMADS15 | - | - | 0.06 | - |
| Pavir.1NG490300.1_x_at | Best-hit-arabi-name=AT2G22540.1 / Best-hit-rice-name=LOC_Os02g52340.1 | AtAGL22, AtSVP / OsMADS22 | - | - | 2.21 | - |
| Pavir.1NG470600.1_s_at | Best-hit-arabi-name=AT2G22540.1 / Best-hit-rice-name=LOC_Os02g52340.1 | AtAGL22, AtSVP / OsMADS22 | - | - | 2.53 | - |
| Pavir.1NG121800.1_x_at | Best-hit-arabi-name=AT5G45890.1 / Best-hit-rice-name=LOC_Os04g01710.1 | AtSAG12 | - | - | - | 7.91 |
| Pavir.4NG227400.1_x_at | Best-hit-arabi-name=AT2G24840.1 / Best-hit-rice-name=LOC_Os06g30810.1 | AtAGL61, AtDIA / OsMADS75 | - | 2.59 | 2.81 | - |
| Pavir.J027700.1_x_at | Best-hit-arabi-name=AT5G45890.1 / Best-hit-rice-name=LOC_Os09g21370.1 | AtSAG12 | - | 2.04 | - | 7.11 |
| Pavir.9NG775900.1_at | Best-hit-arabi-name=AT2G45660.1 / Best-hit-rice-name=LOC_Os10g39130.1 | AtAGL20, AtSOC1 / OsMADS56 | - | - | 2.27 | - |
| Pavir.9KG649500.1_s_at | Best-hit-arabi-name=AT2G22540.1 / Best-hit-rice-name=LOC_Os03g08754.1 | AtAGL22, AtSVP / OsMADS47 | - | - | 4.12 | - |
| Pavir.4NG227400.3_x_at | Best-hit-arabi-name=AT2G24840.1 / Best-hit-rice-name=LOC_Os06g30810.1 | AtAGL61, AtDIA / OsMADS75 | - | 3.31 | 3.35 | 2.06 |
| Pavir.1KG377300.1_at | Best-hit-arabi-name=AT5G57660.1 / Best-hit-rice-name=LOC_Os02g39710.1 | AtCOL5 | - | - | 2.05 | - |
| Pavir.1NG122000.1_x_at | Best-hit-arabi-name=AT5G45890.1 / Best-hit-rice-name=LOC_Os09g21370.1 | AtSAG12 | - | - | - | 8.04 |
| Pavir.4NG172100.4_x_at | Best-hit-rice-name=LOC_Os12g10540.4 | OsMADS13 | 2.10 | - | 2.33 | - |
| Pavir.1NG002100.1_s_at | Best-hit-arabi-name=AT2G45660.1 / Best-hit-rice-name=LOC_Os02g01355.1 | AtAGL20, AtSOC1 | 2.17 | - | - | - |
| Pavir.1NG002100.2_s_at | Best-hit-arabi-name=AT2G45660.1 / Best-hit-rice-name=LOC_Os02g01355.1 | AtAGL20, AtSOC1 | 2.17 | - | - | - |
| Pavir.3KG523600.1_at | Best-hit-arabi-name=AT5G45890.1 / Best-hit-rice-name=LOC_Os03g54130.1 | AtSAG12 | - | 3.90 | - | - |
| Pavir.4NG227400.2_x_at | Best-hit-arabi-name=AT2G24840.1 / Best-hit-rice-name=LOC_Os06g30810.1 | AtAGL61, AtDIA / OsMADS75 | 2.17 | 3.04 | 2.95 | 2.04 |
| Pavir.4NG227400.4_x_at | Best-hit-arabi-name=AT2G24840.1 / Best-hit-rice-name=LOC_Os06g30810.1 | AtAGL61, AtDIA / OsMADS75 | 2.17 | 3.04 | 2.95 | 2.04 |
| Pavir.4NG327000.1_at | Best-hit-arabi-name=AT2G17840.1 / Best-hit-rice-name=LOC_Os06g50330.1 | AtERD7 | 2.26 | - | - | - |
| Pavir.4NG172100.1_x_at | Best-hit-rice-name=LOC_Os12g10540.1 | OsMADS13 | 2.31 | - | 3.14 | 2.43 |
| Pavir.6NG327900.1_s_at | Best-hit-arabi-name=AT4G11880.1 / Best-hit-rice-name=LOC_Os08g41960.1 | AtAGL14 / OsMADS37 | 2.35 | - | - | - |
| Pavir.6NG327900.2_s_at | Best-hit-arabi-name=AT4G11880.1 / Best-hit-rice-name=LOC_Os08g41960.1 | AtAGL14 / OsMADS37 | 2.35 | - | - | - |
| Pavir.3NG137200.1_at | Best-hit-arabi-name=AT4G02380.1 / Best-hit-rice-name=LOC_Os05g29930.1 | AtLEA5, AtSAG21 | - | 9.04 | 0.33 | 6.18 |
| Pavir.9KG649500.2_at | Best-hit-arabi-name=AT2G22540.1 / Best-hit-rice-name=LOC_Os03g08754.2 | AtAGL22, AtSVP / OsMADS47 | 2.43 | - | 3.80 | - |
| Pavir.4NG172100.6_x_at | Best-hit-rice-name=LOC_Os12g10540.2 | OsMADS13 | 2.71 | - | 3.69 | 2.74 |
| Pavir.4KG233600.1_at | Best-hit-arabi-name=AT2G24840.1 / Best-hit-rice-name=LOC_Os06g30810.1 | AtAGL61, AtDIA / OsMADS75 | 3.44 | - | - | - |
| Pavir.6NG327900.3_at | Best-hit-arabi-name=AT4G11880.1 / Best-hit-rice-name=LOC_Os08g41960.1 | AtAGL14 / OsMADS37 | 3.64 | - | - | - |
